# Flexible freestanding MoS_2_-based composite paper for energy conversion and storage

**DOI:** 10.3762/bjnano.10.147

**Published:** 2019-07-24

**Authors:** Florian Zoller, Jan Luxa, Thomas Bein, Dina Fattakhova-Rohlfing, Daniel Bouša, Zdeněk Sofer

**Affiliations:** 1 Department of Chemistry and Center for NanoScience (CeNS), Ludwig-Maximilians-Universität München (LMU Munich), Geschwister-Scholl-Platz 1, 80539 Munich, Germany; 2 Faculty of Engineering and Center for Nanointegration Duisburg-Essen (CENIDE), University of Duisburg-Essen, Lotharstraße 1, 47057 Duisburg, Germany; 3 Department of Inorganic Chemistry, University of Chemistry and Technology Prague, Technická 5, 166 28 Prague 6, Czech Republic; 4 Forschungszentrum Jülich GmbH, Institute of Energy and Climate Research (IEK-1) Materials Synthesis and Processing, Wilhelm-Johnen-Straße, 52425 Jülich, Germany

**Keywords:** flexible composites, hydrogen evolution reaction (HER), lithium ion batteries (LIBs), molybdenum disulfide, nanoarchitectonics, supercapacitors

## Abstract

The construction of flexible electrochemical devices for energy storage and generation is of utmost importance in modern society. In this article, we report on the synthesis of flexible MoS_2_-based composite paper by high-energy shear force milling and simple vacuum filtration. This composite material combines high flexibility, mechanical strength and good chemical stability. Chronopotentiometric charge–discharge measurements were used to determine the capacitance of our paper material. The highest capacitance achieved was 33 mF·cm^−2^ at a current density of 1 mA·cm^−2^, demonstrating potential application in supercapacitors. We further used the material as a cathode for the hydrogen evolution reaction (HER) with an onset potential of approximately −0.2 V vs RHE. The onset potential was even lower (approximately −0.1 V vs RHE) after treatment with n-butyllithium, suggesting the introduction of new active sites. Finally, a potential use in lithium ion batteries (LIB) was examined. Our material can be used directly without any binder, additive carbon or copper current collector and delivers specific capacity of 740 mA·h·g^−1^ at a current density of 0.1 A·g^−1^. After 40 cycles at this current density the material still reached a capacity retention of 91%. Our findings show that this composite material could find application in electrochemical energy storage and generation devices where high flexibility and mechanical strength are desired.

## Introduction

The world’s growing population has a nearly ever-increasing demand for energy. Due to the well-known problem of global warming, there are efforts to shift energy production from burning fossil fuels towards renewable energy sources. However, most of the established renewable energy sources are not suitable to meet the energy consumption requirements today. Hence, energy storage and conversion continues to be an important and urgent issue [1–2].

Lithium ion batteries (LIBs) are one of the most promising energy storage devices, combining high energy density and extremely low self-discharge. Nevertheless, in order to fulfill the (prospective) requirements and to extend their application to large energy storage systems or to the electromobility sector, an improvement in the energy storage capacity is necessary. Layered dichalcogenide materials such as molybdenum sulfide (MoS_2_) are promising candidates for the replacement of the commercial anode material graphite. Apart from this specific application, chalcogenide materials also find numerous applications in various scientific fields [3–5]. During charge/discharge, MoS_2_ undergoes a 4-electron process resulting in a theoretical specific capacity of 669 mA·h·g^−1^, which is almost two times higher than that of graphite (372 mA·h·g^−1^) [6].

However, poor electrical conductivity, capacity fading and large volume changes upon charge and discharge make the commercialization of MoS_2_ in LIBs problematic [6–7]. In order to address this issue, the fabrication of MoS_2_ composites and carbonaceous support materials (such as amorphous carbon [8], carbon nanofibers [7], carbon nanotubes [8] and graphene [9]) has already been demonstrated to be quite attractive. Typically, the electrodes are prepared by mixing these composites as active material with a polymeric binder, conductive carbon and an organic solvent to form a slurry, which is then coated onto a copper foil (current collector). The copper foil and the additives increase the overall weight, which dramatically decreases the gravimetric energy density. These electrodes are not applicable as anodes in flexible batteries due to the loss of contact between the active material and the current collector upon bending deformation [10]. However, there are promising reports on freestanding MoS_2_/carbonaceous composite electrodes which have demonstrated attractive electrochemical performance [9–25].

Beside LIBs, supercapacitors (SCs) are seen as next-generation energy storage devices having a high specific power, fast charge–discharge rate and excellent cycling stability [2]. Freestanding, binder-free electrodes are also of great interest, as they can be used in flexible SCs [26]. In this regard, two-dimensional (2D) graphene has attained significant interest. Nevertheless, materials with higher performance are necessary [26–27]. MoS_2_ is seen, due its layered graphene-analogous structure, as a promising alternative providing a large surface area, which is favorable for double-layer charge storage [27–28]. Moreover, Mo can occupy multiple oxidation states, which enables a pseudo-capacitive charge transfer by insertion of electrolyte ions, such as Li^+^, Na^+^, K^+^ and H^+^ [28–29]. Upon cycling, MoS_2_ sheets can restack resulting in a decreased surface area, which is then followed by poor capacitive performance. Moreover, an appropriate heat management scheme has to be taken into account in real applications as it has been already shown for other nanomaterials [30–31]. Introducing support materials, such as graphene or carbon nanotubes (CNTs) can alleviate these problems and improve the performance of the materials [26].

Another popular related field in the context of energy storage and sustainable energy production is water splitting to produce hydrogen. The best catalysts for the hydrogen evolution reaction (HER) are unequivocally based on platinum and iridium, however the scarcity and the high cost of these materials are tremendous disadvantages for the production of hydrogen on an industrial scale [16]. Hence, it is necessary to develop new catalysts which are abundant, inexpensive and chemically robust [16]. MoS_2_ is again a promising candidate. Theoretical and experimental studies have successfully demonstrated that nanoscale MoS_2_ is more appropriate than the bulk phase equivalent. The surface of the bulk phase mainly consists of thermodynamically more stable basal sites, which are catalytically less active. In contrast, the sulfur edge sites of MoS_2_ are highly catalytically active towards HER [32–34]. However, MoS_2_ possesses only a low intrinsic conductivity, which hinders the charge transport [35]. Using MoS_2_ together with conducting support materials, such as multiwalled carbon nanotubes (MWCNTs) has already been demonstrated to improve the catalytic properties [35].

Herein, we report on the synthesis of a freestanding MoS_2_-based composite paper using a small addition of single-walled carbon nanotubes (SWCNTs) and shear-force milling in *N*-methyl-2-pyrrolidone (NMP). The paper was prepared simply by vacuum filtration of the slurry on top of a filter. The resulting material exhibits high flexibility and combines the high conductivity of SWCNTs and electrochemical potential of MoS_2_. We also show that the material finds use as an anode in LIBs, supercapacitor electrodes and HER catalyst. The application for LIBs seems particularly promising as this composite material requires no additional binders, conductive additives or a current collector.

## Results and Discussion

### Characterization of morphology, composition and mechanical properties

**Figure 1:**
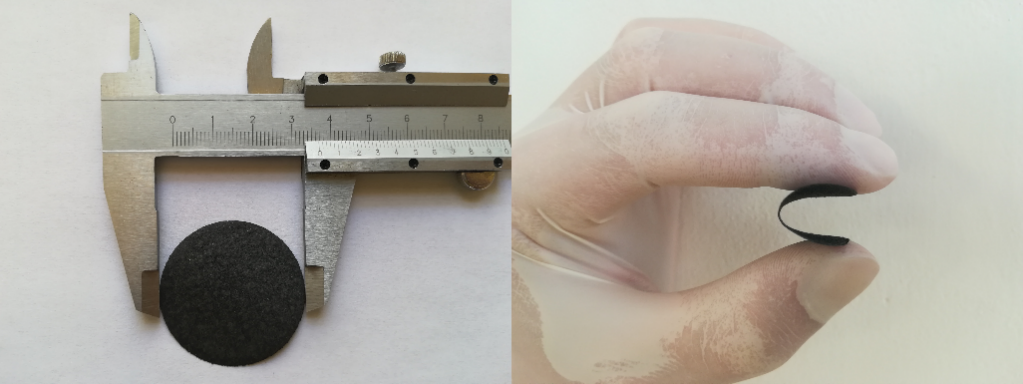
Image of the MoS_2_-based composite paper showing its size and flexibility.

The synthesized composite material based on MoS_2_ and SWCNTs was prepared by shear-force milling of MoS_2_ powder with SWCNTs. We then prepared a paper-like material by filtration of the mixture on top of a filter. The self-assembled material was denoted as MoS_2_-based composite paper. A picture of the composite paper is shown in Figure 1.

**Figure 2:**
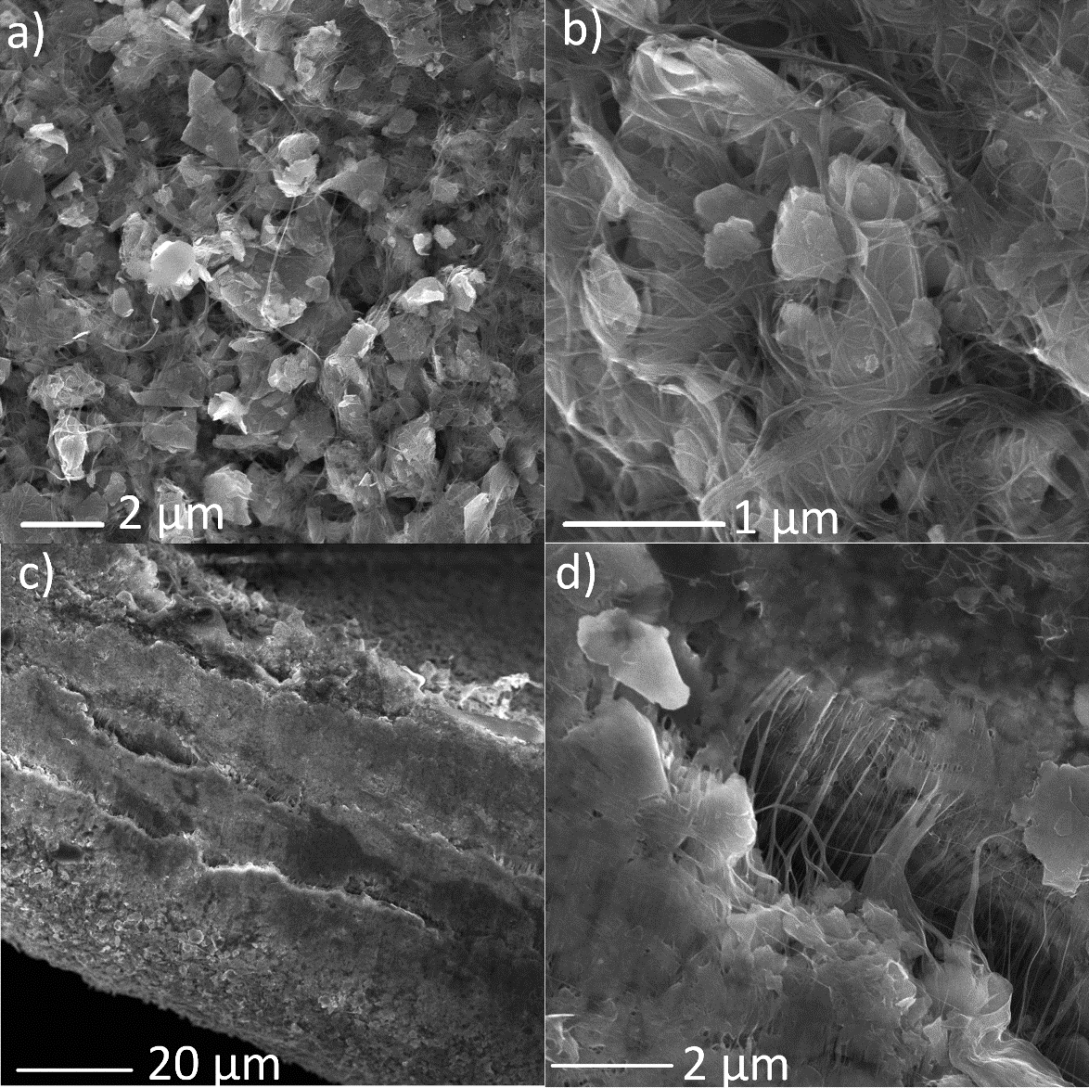
SEM micrographs of (a,b) plane and (c,d) cross-section images of the composite paper at different magnifications.

We investigated the morphology of this compound material by scanning electron microscopy (SEM) with results shown in Figure 2. The morphology images of the top side of the composite paper (Figure 2a and 2b) show a homogeneous distribution of SWCNTs among the MoS_2_ sheets. SEM micrographs of the cross-section (Figure 2c and 2d) also illustrate that the SWCNTs significantly contribute to the flexibility and mechanical strength of the composite as they hold individual sheets together.

We also performed the composition characterization by energy-dispersive spectroscopy (EDS). The elemental composition maps (Supporting Information File 1, Figure S1) revealed a homogeneous distribution of elements. We have also identified (Supporting Information File 1, Table S1) that there was about 2.1 wt % of iron in the sample. This contamination originates from the carbon nanotubes, where iron usually serves as a catalyst for their growth [36].

**Figure 3:**
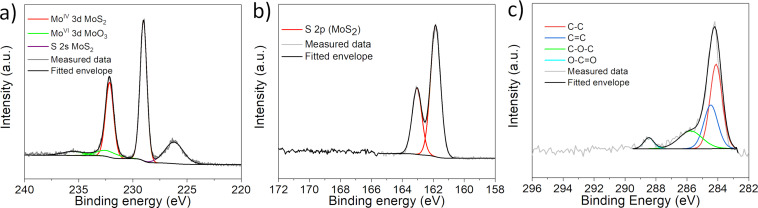
Core-level X-ray photoelectron spectra of a) Mo 3d region, b) S 2p region, and c) C 1s region.

X-ray photoelectron spectroscopy (XPS) was used to track the degree of degradation of the MoS_2_ sheets. Components originating from MoS_2_ and MoO_3_ were identified in the core-level Mo 3d spectrum (Figure 3). The positions of the individual components are in agreement with previous reports for MoS_2_ and MoO_3_ [37–38]. The deconvolution revealed that the MoO_3_ content was about ≈12 atom %. This degree of oxidation is lower than in the case of chemically exfoliated MoS_2_, which is possibly due to a slightly lower degree of exfoliation [39]. Additionally, no oxidation was observed for sulfur as only states originating from sulfides were identified in the S 2p spectrum (Figure 3b) [40].

The chemical states of the SWCNTs could not be precisely determined due to the overlap with adventitious carbon. However, the conditions used during our experiment were highly unlikely to cause any chemical changes in the SWCNTs.

Additionally, the mechanical properties of prepared MoS_2_-based composite paper were evaluated. The tensile strength and tensile ductility are important material parameters that influence material’s final applicability. The tensile strength of the prepared material reached a value of 3.02 MPa while tensile ductility was 7.74%. It should be mentioned that the preparation of paper solely from MoS_2_ sheets is not possible since there is no material holding the individual MoS_2_ sheets together. On the other hand, paper made of only SWCNT possesses a tensile strength of 5.95 MPa and tensile ductility of 2.45%. Therefore, incorporation of MoS_2_ sheets into SWCNT paper results in decreased tensile strength and increased tensile ductility and the as-prepared MoS_2_-based composite paper is able to undergo significant plastic deformation before rupture in the material occurs.

### Electrochemical performance of freestanding MoS_2_-based composite paper

First, we tested the MoS_2_-based composite paper for applications in supercapacitors (SCs). The capacitance was measured by a chronoamperometry technique in KCl solution (1 M) using different charging–discharging current densities (1–5 mA·cm^−2^) in a potential range determined by cyclic voltammetry (CV) shown in Supporting Information File 1, Figure S2. The CV curves demonstrate the rectangular shape pointing out the electric double-layer capacitance as the origin of capacitive behavior. The capacitance *C* in units of mF·cm^−2^ was calculated from the value of discharging current *I*, discharging time *t*, maximal voltage *U* and the area of the electrode that comes into contact with the electrolyte solution *S*. The calculation was performed using equation: *C* = (2·*I*) / (*S*·*U*/*t*).

**Figure 4:**
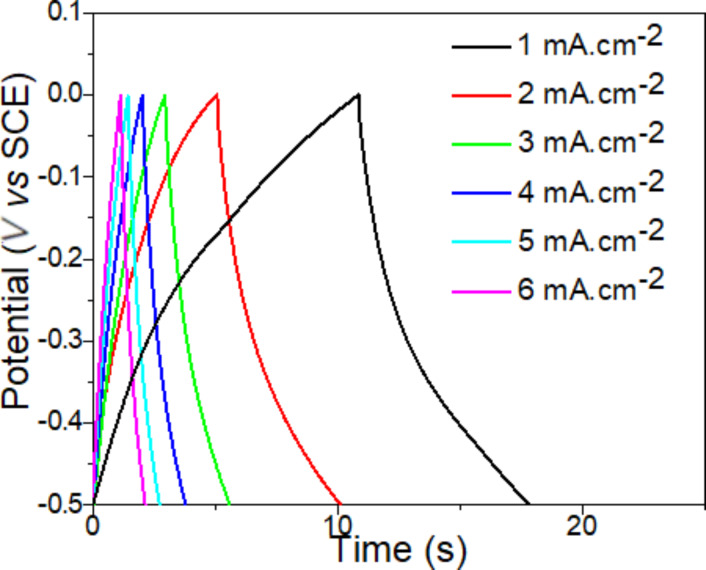
Charging–discharging curves of MoS_2_-based composite paper obtained by chronoamperometry in 1M KCl solution using different current densities.

The calculated values are summarized for each discharging current in Table 1. Charging–discharging curves of MoS_2_-based composite paper obtained using the chronoamperometry measurement are shown in Figure 4. We also compared the capacitance of our composite MoS_2_-based composite paper with other reported materials (see Table 2). The capacitance of our composite material exhibits a competitive value compared to other materials reported in the literature.

**Table 1:** Capacitance of MoS_2_-based composite paper measured using various discharging current densities.

Discharging current density (mA·cm^−2^)	1	2	3	4	5	6

Capacitance (mF·cm^−2^)	70	40	35	33	29	28

**Table 2:** Performance of various electrode materials in the literature compared to this work.

Ref.	Electrode material	Electrolyte	Capacitance (mF·cm^−2^)

this work	MoS_2_-based composite paper	KCl	33
[41]	interdigital MWCNT electrode	PVA/KOH	107.3
[42]	CNT	PVDF-HFP/EMIMTFSI	2.88
[43]	graphene	PVA-H_2_SO_4_	2.32
[44]	graphene–CNT	KCl	2.8

**Figure 5:**
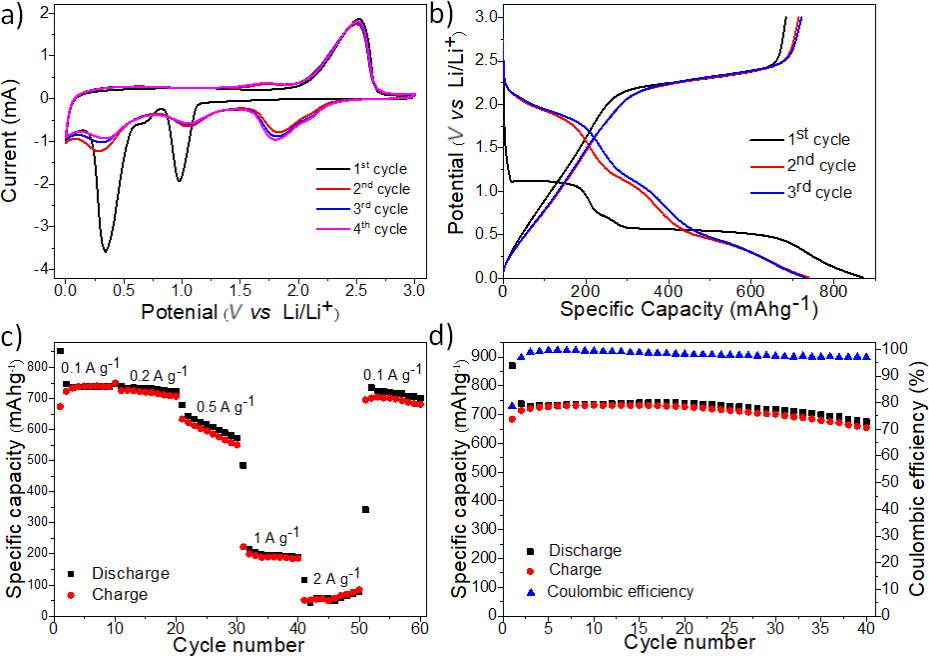
Electrochemical analysis of the freestanding MoS_2_-based composite paper. (a) CV curves at a scan rate of 0.1 V·s^−1^, (b) discharge/charge voltage profiles at 0.2 A·g^−1^, (c) reversible capacities at different current densities ranging from 0.1 to 2.0 A·g^−1^ and (d) cycling performance and coulombic efficiency at 0.2 A·g^−1^.

The freestanding MoS_2_-based composite paper was also tested as an anode material for LIBs. Hence, the MoS_2_-based composite paper was used directly as an anode without any binder, carbon additive or a Cu-foil current collector. Figure 5a shows the first four cycles of the cyclic voltammetry (CV) curves of the MoS_2_-based composite paper. The measurements were performed at a scan rate of 0.1 mV·s^−1^ in the voltage range of 0.01–3.0 V vs Li/Li^+^. In the initial cathodic scan, two dominant reduction peaks at around 1.0 and 0.3 V are detectable (Figure 5a). The first is associated with the insertion of lithium ions into the van der Waals spaces between the MoS_2_ layers forming Li*_x_*MoS_2_ accompanied by a phase transformation from trigonal prismatic (2H) to octahedral (1T) (see the following Equation 1) [8,20–21]. The peak at ≈0.3 V corresponds to the conversion of the previously formed Li*_x_*MoS_2_ into metallic Mo and LiS_2_ (see the following Equation 2) and the decomposition of the electrolyte followed by the formation of a solid electrolyte interphase (SEI) layer [18,20]. The prominent anodic peak at ≈2.5 V results from the conversion of Li_2_S to sulfur and lithium ions (see the following Equation 3) [20]. During the following discharge cycles the two peaks at ≈1.0 and ≈0.3 V diminish and three new reduction peaks at around 1.8, 1.1 and 0.3 V appear, which can be ascribed to the following reactions (Equations 1–3):

[4] $${\text{2Li}}^{+}+\text{ S }+\;2e^{\text{-}}\to {\text{ Li}}_{\text{2}}\text{S}$$

[1] $${\text{MoS}}_{\text{2}}+x{\text{Li}}^{+}+xe^{-}\to {\text{ Li}}_{x}{\text{MoS}}_{\text{2}}$$

[2] $${\text{Li}}_{x}{\text{MoS}}_{\text{2}}+\;\left(4\;–x\right){\text{Li}}^{+}+\;\left(4\;–x\right)e^{-}\to \text{ Mo }+{\text{ 2Li}}_{\text{2}}\text{S}$$

[3] $${\text{Li}}_{\text{2}}\text{S }\to \;2{\text{Li}}^{+}+\text{ S }+\;2{\text{e}}^{\text{-}}$$

Hence, the reduction peak at ≈1.8 V and the oxidation peak at 2.5 V form together a reversible redox couple [20]. Starting with the second cycle, a shallow oxidation peak arises at ≈1.7 V which can be attributed to the partial oxidation of metallic Mo to MoS_2_ [21,45].

Moreover, the electrochemical performance of the MoS_2_-based composite paper is evaluated by galvanostatic discharge/charge measurements as well. The lithiation and delithiation plateaus (Figure 5b) obtained at a current density of 0.2 A·g^−1^ are consistent with the reduction and oxidation peaks gathered from the CV measurement.

In the first cycle, discharge and charge capacities of 870 and 684 mA·h·g^−1^ were obtained, respectively. This corresponds to a coulombic efficiency of 79%, as shown in Figure 5b and Figure 5d. The irreversible initial capacity loss is mainly attributed to the formation of the SEI layer [8,17,20]. During the subsequent cycles, coulombic efficiencies of ≤97% are reached, which implies a good cycling reversibility (Figure 5d). After 40 cycles a specific capacity of 675 mA·h·g^−1^ is reached equaling a capacity retention of 78% compared to the initial cycle or 91% when compared to the second cycle. It should be noted that for the calculation of the specific capacitiy the total mass of the freestanding MoS_2_-based composite paper electrode was used.

Moreover, the rate performance of the freestanding MoS_2_-based composite paper electrodes was further investigated (Figure 5c). The composite delivers 740, 721, 596, 190 and 49 mA·h·g^−1^, at current rates of 0.1, 0.2, 0.5, 1 and 2 A·g^−1^, respectively. The slightly increasing capacity during the 2 A·g^−1^ step may be attributed to the high current rate activation of new Li^+^ storage sites, originating from the opening up of blocked ends of SWCNTs [8]. Interestingly, when the current density was set back to 0.1 A·g^−1^ the capacity reached 681 mA·h·g^−1^ (80th cycle) equaling a capacity retention of 91% compared to the second cycle, also confirming the high structural stability of the freestanding MoS_2_-based composite paper. The kinetic analysis of the MoS_2_-based composite paper is described in Supporting Information File 1, Figure S2.

**Figure 6:**
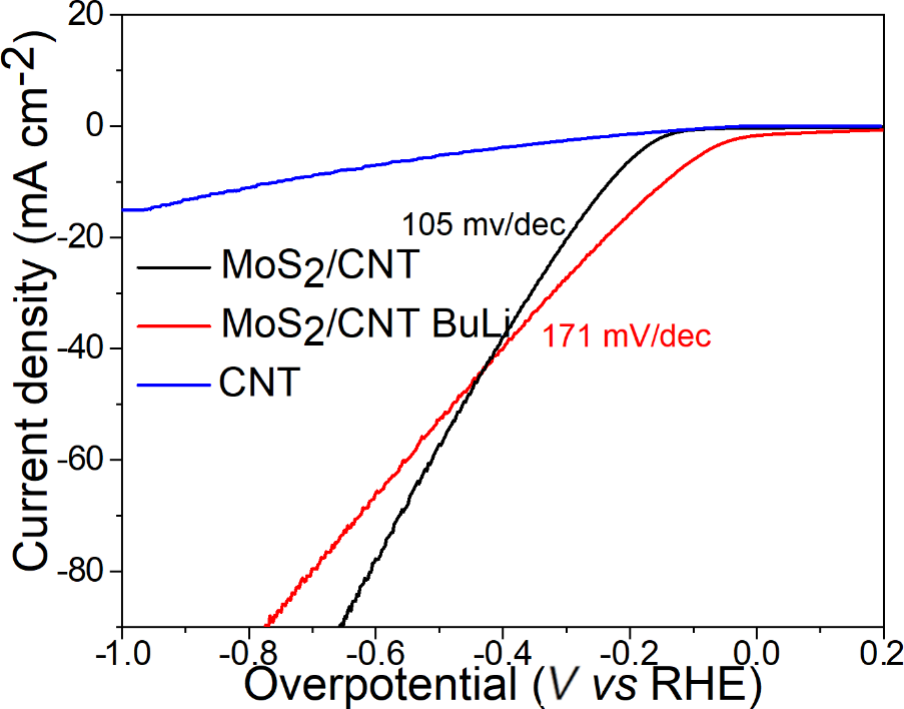
Linear sweep voltammetry curves for the hydrogen evolution reaction measurements in 0.5 M H_2_SO_4_, 2 mV·s^−1^.

Finally, we tested the MoS_2_-based composite paper as a catalyst for the hydrogen evolution reaction (HER). The results are shown in Figure 6. Apart from the pristine material, we also treated the paper with *n*-butyllithium (BuLi) solution to introduce new active sites in the form of edge sites as well as defects. The pristine as-prepared material exhibited an onset potential of about −0.195 V vs RHE. On the other hand, BuLi-exfoliated MoS_2_-based composite paper showed improved activity with an onset potential of about −0.095 V vs RHE. These differences clearly demonstrate that new sites were indeed introduced by the treatment. However, Figure 6 also demonstrates that BuLi-treated samples exhibited substantially higher (171 mV/dec) Tafel slope values than the pristine sample (105 mV/dec). This discrepancy could be caused by a loss of proper connection between the MoS_2_ sheets and SWCNTs and a decrease in conductivity. This claim is supplemented by the fact that the paper material exhibited lower flexibility than the original one.

## Conclusion

Using shear-force milling, we prepared a freestanding MoS_2_-based composite paper material. This method is very simple and takes advantage of the fact that a paper-like material is self-assembled on top of a filter during vacuum filtration. The reported material exhibits high structural integrity and flexibility. The composite was tested in various electrochemical applications covering supercapacitors, anodes in lithium ion batteries and hydrogen evolution catalysis. In terms of supercapacitors, our material exhibits a capacitance of 33 mF·cm^−2^ at a current density of 1 mA·cm^−2^. This value is competitive with other reported materials based on carbon nanomaterials. This material can also be used as a hydrogen evolution reaction catalyst. The as-prepared materials exhibits an onset potential of approximately −0.195 V vs RHE and is capable of reaching current densities as high as 100 mA·cm^−2^. Additionally, we treated the material with *n*-butyllithium to further enhance the HER activity. The resulting material exhibited a lower onset potential, however, the exfoliation of MoS_2_ sheets resulted in a loss of connection between the MoS_2_ sheets and SWCNTs. Ultimately, this led to a decrease in the conductivity, and consequently, substantial increase in the Tafel slope value. The MoS_2_-based composite paper was also tested as a freestanding anode in LIBs without additives such as binders or conductive agents. After the initial loss of specific capacity due to the formation of the solid electrolyte interface, the composite delivers a specific capacity of 740 mA·h·g^−1^ at 0.1 A·g^−1^. Moreover, the material retains 91% of its capacity after 40 cycles. A high capacity retention was also observed after the rate performance tests. These findings show that the reported material is also promising for application in flexible batteries.

## Experimental

### Materials

MoS_2_ was purchased from Alfa Aesar, TUBALL^TM^ SWCNTs were purchased from OCSiAl, and *N*-methyl-2-pyrrolidone (NMP) was purchased from Sigma-Aldrich.

The PuriEL electrolyte (1.15 M LiPF_6_ in ethylene carbonate/ethyl methyl carbonate/dimethyl carbonate (EC/EMC/DMC) = 2:2:6 v/v + 1.0 wt % fluoroethylene carbonate (FEC), *soul*brain MI) and lithium metal (Rockwood) were used as received.

### Preparation of MoS_2_-based composite paper

125 mg of MoS_2_ powder and 12.5 mg of SWCNTs were added to 80 mL of Ar-purged NMP. The suspension was then exfoliated under Ar atmosphere for 2 h at 16,000 rot/min using a high-energy shear-force disperser. After that, the mixture was vacuum-filtered on top of a nylon filter and washed with methanol several times. The use of methanol significantly shortens the time necessary for drying. The resulting material was then self-assembled into the form of a paper-like material. After drying under vacuum, the material was directly used.

### Battery assembly and electrochemical measurements

The freestanding MoS_2_-based composite paper was cut into round disks with a diameter of 18 mm (254.5 mm^2^). They were directly used as an anode in ECC-PAT-Core (EL-Cell) battery test cells assembled in an argon-filled glove box using lithium metal both as the counter and reference electrode and an EL-CELL ECC1-01-0011-A/L glass fiber membrane as a separator. The used electrolyte consisted of a commercial mixture of 1.15 M LiPF_6_ in EC/EMC/DMC at a 2:2:6 v/v and 1.0 wt % FEC.

The electrochemical measurements were performed at room temperature using an Autolab potentiostat/galvanostat (PGSTAT302N) with a FRA32M module or an Autolab multipotentiostat M101 with a 8AUT.M101 module operated with Nova 1.11 software. The cyclic voltammograms were recorded in a potential range of 0.01–3.0 V vs Li/Li^+^ using a scan rate ranging from 0.05 mV·s^−1^ to 1 mV·s^−1^. The cells were charged and discharged galvanostatically at different *C* rates (0.1 to 2 A·g^−1^) in a voltage range of 0.01–3.0 V vs Li/Li^+^.

### Supercapacitors

A disk with diameter of 14 mm was cut from the vacuum-assembled MoS_2_-based composite paper material. Then it was placed into an electrochemical holder (InRedox, USA) which was placed in the middle of platinum basket (counter electrode). A saturated calomel reference electrode was used as the reference electrode and the measurements were performed in 1 M KCl solution. The exposed area was a disk with 0.94 mm diameter. In order to measure the charge–discharge curves, several charging–discharging currents (1–5 mA·cm^−2^) were used to charge the material to −0.8 V vs SCE.

### Hydrogen evolution reaction

For the HER measurements, the pristine sample was placed in an electrochemical holder (InRedox, USA) which was inserted into the 0.5 M H_2_SO_4_ electrolyte with SCE and a carbon rod as the reference and counter electrodes, respectively. The scan rate was 2 mV·s^−1^. For the *n*-butyllithium treated sample, the foil was left in *n*-butyllithium solution (2.5 M solution) for several days under inert Ar atmosphere. After that, water was added to the solution. The foil was then dried and used.

### Characterization

The morphology was investigated using scanning electron microscopy (SEM) with a FEG electron source (Tescan Lyra dual beam microscope). The elemental composition and mapping were performed using an energy dispersive spectroscopy (EDS) analyzer (X-MaxN) with a 20 mm^2^ SDD detector (Oxford Instruments) and AZtecEnergy software. A 10 kV beam was used for the measurements.

High-resolution X-ray photoelectron spectroscopy (XPS) was performed using an ESCAProbeP spectrometer (Omicron Nanotechnology Ltd, Germany) with a monochromatic aluminum X-ray radiation source (1486.7 eV). Wide-scan surveys of all elements were performed (0–1000 eV, step 0.5 eV) with subsequent high-resolution scans of the C 1s, S 2p and Mo 3d regions with a step of 0.05 eV.

The dynamic mechanical analysis was measured on a DMA DX04T (by RMI, Czech Republic) device. A sample with dimensions 7.600 mm (width), 0.173 mm (thickness) and 10.200 mm (active length) was loaded with a tensile longitudinal sinusoidal deformation with the amplitude of 0.02 mm and pretension of 0.03 mm. The temperature range was 20 to 200 °C with a heating rate of 2 °C·min^−1^ in air atmosphere. From the results, the values of the moduli and loss factor were evaluated as the second-order sliding average.

## Supporting Information

File 1 Additional experimental results.
