# Supplementary material for: Flexible freestanding MoS2-based composite paper for energy conversion and storage
Source: Beilstein J Nanotechnol. 2019 Jul 24;10:1488–96. doi: 10.3762/bjnano.10.147 (PMC6664410; doi:10.3762/bjnano.10.147)
Supplement: File 1 — Additional experimental results. [file Beilstein_J_Nanotechnol-10-1488-s001.pdf]

BEILSTEIN JOURNAL OF NANOTECHNOLOGY

Supporting Information

for

Flexible freestanding MoS_2-based composite paper for energy conversion and storage

Florian Zoller, Jan Luxa, Thomas Bein, Dina Fattakhova-Rohlfing, Daniel Bouša and Zdeněk Sofer

Beilstein J. Nanotechnol. **2019,** *10,* 1488–1496. doi:10.3762/bjnano.10.147

Additional experimental results

License and Terms: This is a supporting information file under the terms of the Creative Commons Attribution License (http://creativecommons.org/licenses/by/4.0). Please note that the reuse, redistribution and reproduction in particular requires that the authors and source are credited.
The license is subject to the *Beilstein Journal of Nanotechnology* terms and conditions: (https://www.beilstein-journals.org/bjnano)

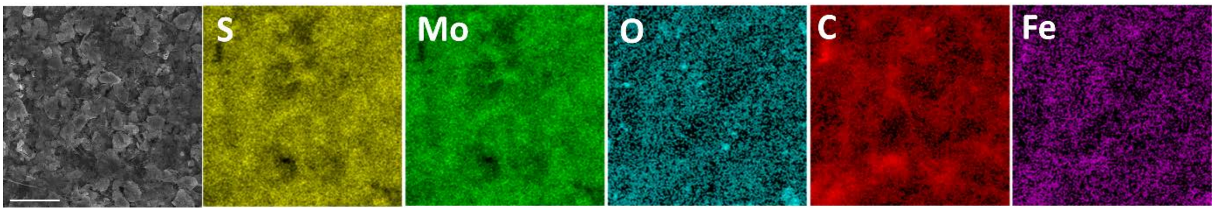

Figure S1. Elemental distribution maps of the MoS_2/SWCNT-paper.

Table S1. Elemental composition obtained from SEM-EDS.

Sample	S wt %	Mo wt %	O wt %	C wt %	Fe wt %
**MoS_2 based composite paper**	29.7	40.9	2.1	25.1	2.1

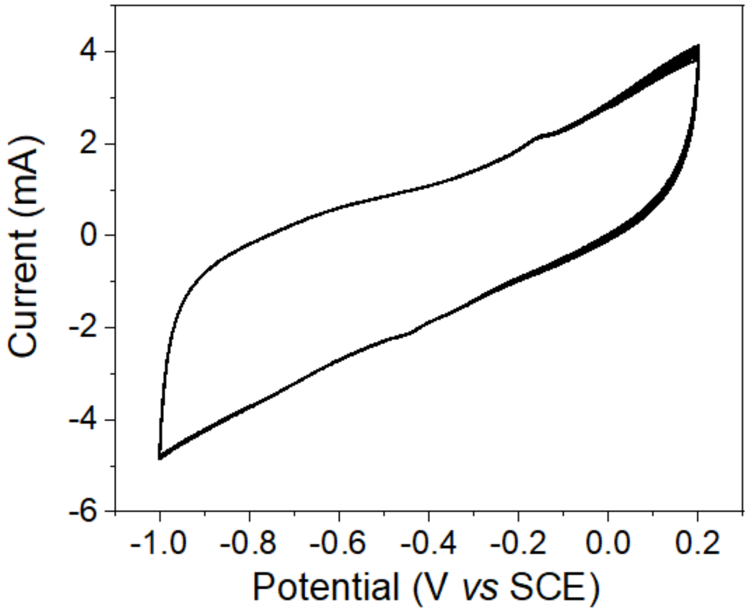

Figure S2. Cyclic voltammograms of MoS_2 based composite paper in 1 M KCl. Scan rate 100 mV s^{-1}.

Kinetics analysis

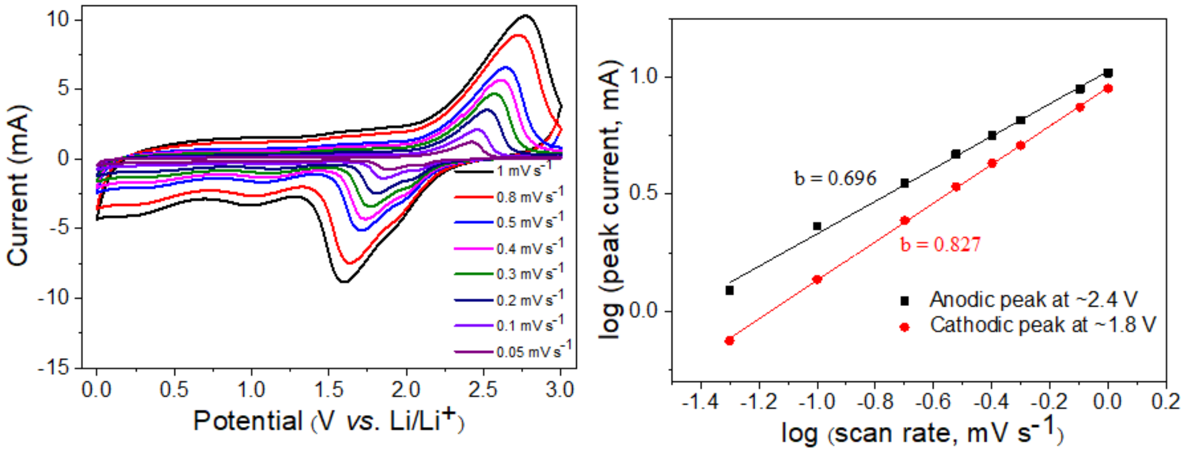

Figure S3. Kinetic analysis of the freestanding MoS_2 based composite paper. (a) CV curves at different scan rates, (b) relationship between logarithmic peak current (cathodic peak at ~1.8 V and anodic peak at ~2.4 V) and logarithmic scan rates.

CV measurements were conducted at different scan rates ranging from 1 to 0.05 mV s^{-1} to gain insights into the reaction kinetics (Figure S2). The degree of capacitive effect can be qualitatively analyzed according to the power law:

$$i = av^{b} \qquad \text{(S1)}$$

which relates the measured peak current (*i*) to the scan rate (*v*), *a* and *b* are adjustable parameters [1]. The *b* value can be determinate by fitting log(*i*) *versus* log(*v*) (Figure S2) [2]. In general, *b* = 1 entails a capacitive surface-limited process, while *b* = 0.5 implies a semi-infinite linear diffusion-controlled mechanism [S1-S3]. For the cathodic peak at ~1.8 V and the anodic peak ~2.4 V *b* values of 0.827 and 0.696 were calculated, respectively. Those values indicate a higher capacitive contribution in the case of the reduction process compared to the oxidation analogue.

References

S1. Xia, S.; Wang, Y.; Liu, Y.; Wu, C.; Wu, M.; Zhang, H. *Chem. Eng. J.* **2018**, *332*, 431-439.

S2. Wu, J.; Lu, Z.; Li, K.; Cui, J.; Yao, S.; Ihsan-ul Haq, M.; Li, B.; Yang, Q.-H.; Kang, F.; Ciucci, F.; Kim, J.-K. *J. Mater. Chem. A* **2018**, *6*, 5668-5677, 10.1039/C7TA11119C.

S3. Wang, G.; Zhang, J.; Yang, S.; Wang, F.; Zhuang, X.; Müllen, K.; Feng, X. *Adv. Energ. Mater.* **2017**, *8*, 1702254.
